# Improved surgical resection of metastatic pancreatic cancer using uPAR targeted *in vivo* fluorescent guidance: comparison with traditional white light surgery

**DOI:** 10.18632/oncotarget.27220

**Published:** 2019-10-29

**Authors:** Karina Juhl, Anders Christensen, Niclas Rubek, Kirstine Kim Schmidt Karnov, Christian von Buchwald, Andreas Kjaer

**Affiliations:** ^1^ Department of Clinical Physiology, Nuclear Medicine & PET, Rigshospitalet, University of Copenhagen, Copenhagen, Denmark; ^2^ Cluster for Molecular Imaging, Department of Biomedical Sciences, University of Copenhagen, Copenhagen, Denmark; ^3^ Department of Otolaryngology, Head and Neck Surgery and Audiology, Rigshospitalet, University of Copenhagen, Copenhagen, Denmark; ^*^ Kirstine Kim Schmidt Karnov sadly passed away before publishing of this article. We will miss her and our thoughts are with her family

**Keywords:** optical imaging, near-infrared imaging (NIR), NIR-I, urokinase plasminogen activator receptor (uPAR), cancer

## Abstract

Pancreatic cancer remains one of the deadliest cancers. The five-year survival rates have been reported as 3%. Radical surgical tumor resection is critical for improved outcome and the low survival rate for pancreatic cancer is due to lack of other effective treatments and here optical guided surgery could be a solution for better surgical outcome. In the present study, we targeted the urokinase plasminogen activator receptor (uPAR) with a peptide conjugated with the fluophore ICG (ICG-Glu-Glu-AE105) for optical imaging. In the first part of the study we aimed to validate ICG-Glu-Glu-AE105 for resection of the primary tumor and metastases in an orthotopic human xenograft pancreatic cancer model. In the second part of the study we aimed to investigate if fluorescent-guided imaging could locate additional metastases following conventional removal of metastasis under normal white light surgery.

Our study showed that ICG-Glu-Glu-AE105 was an excellent probe for intraoperative optical imaging with a mean tumor-to-background ratio (TBR) for the primary tumor of 3.5 and a TBR for the metastases of 3.4. Further, a benefit using intraoperative fluorescent guidance yielded identification of an additional 14% metastases compared to using normal white light surgery. In 4 of 8 mice there were identified additional metastases with uPAR optical imaging compared to white light.

In conclusion, the uPAR-targeted optical probe ICG-Glu-Glu-AE105 enables intraoperative optical cancer imaging, including robotic surgery, and may be a benefit during intended radical resection of disseminated pancreas cancer by finding more metastasis than with traditional white light surgery.

## INTRODUCTION

Pancreatic cancer remains one of the most lethal cancer types and is the fourth leading cause of cancer-related deaths the United States [1, 2]. Patients are often diagnosed in an advanced stage of disease where radical tumor resection is no more an option. Thus 80% of the diagnosed patients are treated with a palliative intention only. Despite intense research in pancreatic cancer over the past decade, the 5-year survival rate remains at only 3%. In 20% of the patients that are suitable for surgery, the vast majority experience relapse, and surgery only increases the median survival with 9.1 months from 3.5 to 12.6 months [3]. Hence, improved and more precise surgical procedures are urgently needed. A new technique that has emerged in the surgical field in recent years is real-time intraoperative optical imaging. This technology enables the surgeon to locate and delineate tumor lesions guided by a targeted fluorescent signal using a near-infrared (NIR) camera [4, 5]. Until now passive retention in tumor lesions due to the enhanced permeability and retention (EPR) effect of larger molecules as indocyanine green (ICG) has been used to visualize tumor lesions [6]. This works well in theory, but the EPR effect has shown great heterogeneity in different tumors and therefore it cannot be applied as a global cancer marker [7]. Thus, cancer specific probes are needed for better guidance across different types of cancer. Clinical trials investigating new optical compounds in cancer surgery are currently ongoing. Accordingly, the OTL38 probe targeting the folate receptor has recently been through a Phase I study [8] and has entered Phase II studies [4]. Likewise, an EGFR targeting antibody cetuximab labelled with the fluophore IRDye800 has also shown promising results in phase I and II studies [9]. However, the heterogeneous expression of biomarkers in different cancer types highlights the need for new targets and probes for imaging of a broader group of cancer patients.

Urokinase-type plasminogen activator receptor (uPAR) is a membrane-bound protein highly expressed in most cancer types including pancreatic cancer [10], oral cancer [11, 12], glioblastomas [13], breast cancer [14], and colorectal cancer [15]. In pancreatic cancer, uPAR is often expressed at the invasive front of the tumor and in the immediately surrounding activated stromal component [16]. Accordingly, uPAR is an ideal target for intraoperative imaging for delineation of cancer tissue. As uPAR is a well-known marker for tumor invasion and aggressive disease, our group previously developed uPAR PET tracers using the same peptide (AE105) [17, 18] that are currently undergoing Phase II testing in several types of cancer. Taking offset in an uPAR-targeting platform, we therefore further developed our targeting peptide into an optical imaging probe using ICG as fluorophore [19]. This strategy offers a unique opportunity to combine pre-operative PET imaging, for selection of patients eligible for intraoperative use of uPAR optical imaging. We have previously validated the rational of combined uPAR-directed PET and optical imaging in a preclinical study [20]. Preoperative uPAR PET imaging provides a tool to better evaluate the extent of the tumor resection needed and the expression level of the receptor, which is vital when planning surgery. Using uPAR guided optical surgery improves the possibility of obtaining surgical radicality in regards to margins and finding additional metastasis and thereby improve long term survival.

The aim of the present study was therefore to investigate if the use of the uPAR-targeted optical probe ICG-Glu-Glu-AE105 in an orthotopic xenograft model of human pancreatic cancer could improve the surgical outcome by detecting additional cancer lesions not found during white light surgery.

## RESULTS

**Figure 1:**
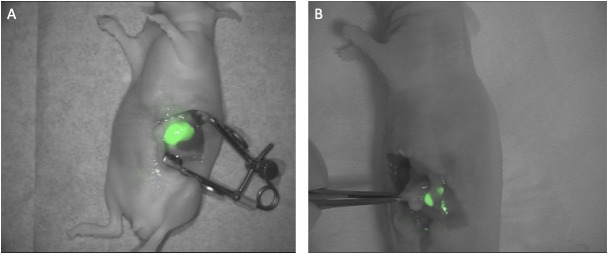
Fluorescent images of pancreatic tumors. (**A**) Representative fluorescent image of a primary tumor developed after inoculation of the cell line BxPC3. luc2 in the tail of the pancreas. The image was taken 15 h post iv. injection of ICG-Glu-Glu-AE105 with the clinically approved fluorescent camera Fluobeam800^®^ (**B**). Several days after removal of the primary tumor small tumor metastases developed in the peritoneal cavity and were clearly visible by fluorescent imaging after iv. injection of ICG-Glu-Glu-AE105. In both images the fluorescent image is processed in Image J and merged with a white light image.

In the first part of the study, where the aim was to show the feasibility of the probe in an orthotopic pancreatic human xenograft cancer model, both the primary tumor and metastatic lesions were detected by the use of the fluorescent signal *in vivo*. Tumor cells were luciferase (luc) transfected and could thus be detected with not clinically translatable bioluminiscence (BLI) as a reference. Imaging of the primary tumor showed a clear delineation of the tumor lesion located in the pancreas with a very low background signal (Figure 1A). During imaging of the metastatic lesions, all foci visible by the naked eye were also detected with the fluorescent signal. In addition, several foci, were only detected by the fluorescent signal (Figure 1B). Lesions down to less than one millimeter, that were not possible to detect with white light, could only be detected with fluorescent imaging. Tumor-to-background (TBR) values for the primary tumors and the metastatic lesions were 3.5 (95% confidence interval (Cl): 3.3; 3.7) and 3.4 (Cl: 3.1; 4.0), respectively (Table 1).

**Table 1:** Tumor-to-background (TBR) values for primary tumor and metastases

	Mean TBR	95% confidence interval
Primary tumor	3.5 (*n* = 5)	3.3; 3.7
Metastases	3.4 (*n* = 9)	3.1; 4.0

Some metastases were down to 1 mm^3^ and still clearly visible. Tumor to background values.

Supplementary Video 1 demonstrates the feasibility of the probe to localize millimeter foci. A metastasis in the abdominal region was easily identified with the Fluobeam camera and then resected by the surgeon. In this situation a small residual deposit was left behind during the resection but was clearly picked up by the camera and enabled the surgeon to perform a complete radical resection by removing the foci detected.

**Figure 2:**
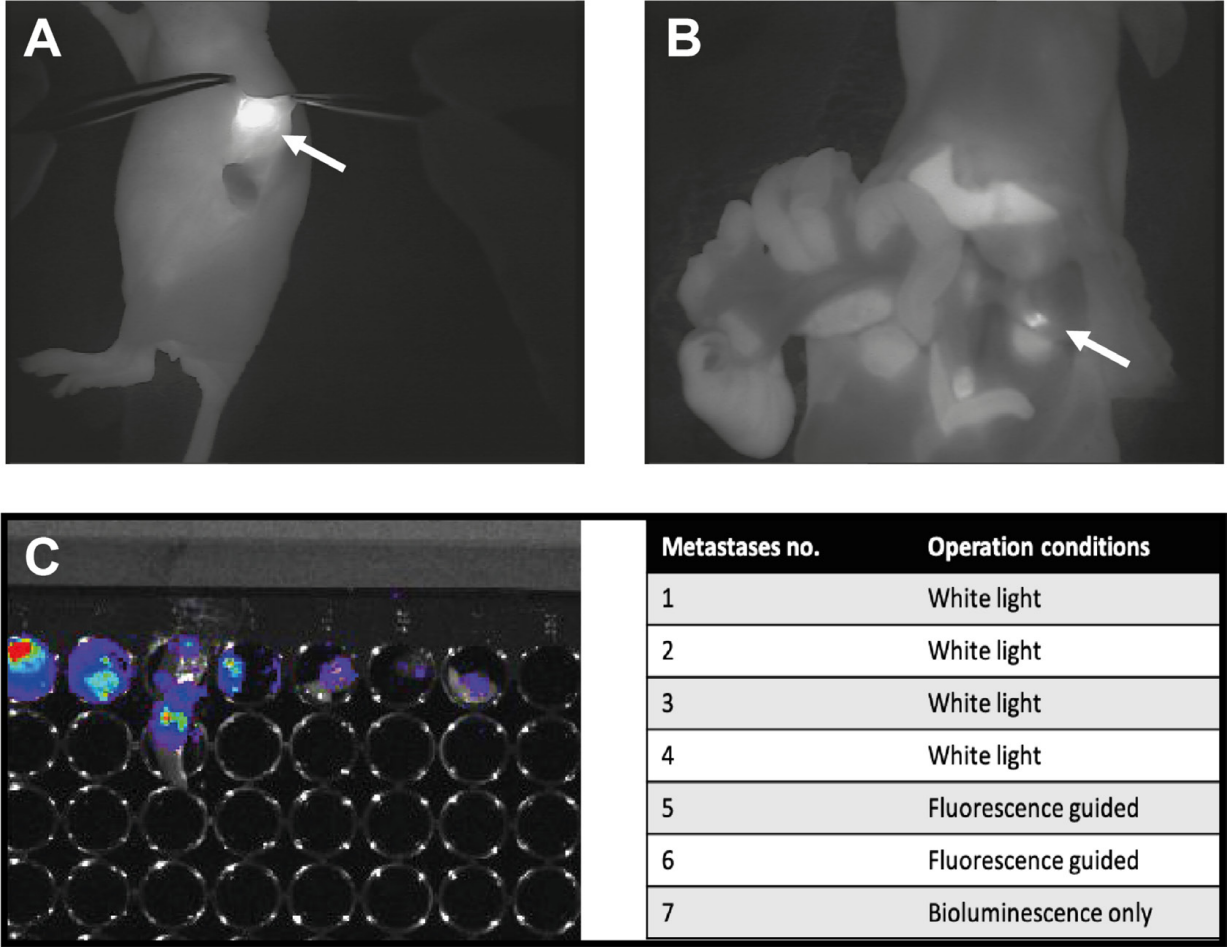
Presentation of one of the mice enrolled in the study part II where comparison of white light surgery and fluorescent guided surgery was the aim. (**A**) Fluorescent image of orthotopically placed primary pancreas tumor 15 h post injection of ICG-Glu-Glu-AE105. (**B**) Fluorescent image of a metastases left behind after surgery with white light only. This metastases was detected with the fluorescent camera Fluobeam800^®^ only and was not visible during white light operation. (**C**) Bioluminescence was used as the gold standard for verification of the presence of tumor cells. All suspected foci (white light and fluorescent) were investigated for presence of tumor cells from a bioluminescence image. (**D**) Table overview of suspected tumor foci found throughout the surgery of a representative mouse. No. 1–4 were found under normal surgery condition, no. 5–6 were found after turning the fluorescent camera on. No. 7 was found only by imaging the animal after ended surgery with bioluminescence.

The second part of the study aimed to evaluate if optical imaging could identify additional metastases after all metastases visible with white light had been removed (Table 2). On a *lesion-basis,* a total of 43 positive metastases identified with bioluminescence (mean = 5.4 (range: 3–7) were present in the 8 mice. Of these 43 metastases, 29 metastases were found without fluorescent guidance (white light), and an additional 6 metastases were identified only with the Fluobeam^®^800 camera (Figure 2A, 2B). Finally, an additional 8 metastases were found only with non-translatable bioluminescence imaging. On an *individual-basis*, in 4 of the 8 mice (50%) there were identified additional metastases with uPAR optical imaging compared to white light. Hence, in 50% of the mice the surgeon operated in white light there would have been left behind metastases that could be found with uPAR optical imaging.

**Table 2:** Number of metastasis found during surgery

	Number of metastases/number of mice	Percentage of total number of metastasis found	95% confidence interval
**Metastasis**			
Found with white light	29	67%	52; 81%
Additionally found with FGS	6	14%	5; 28%
**Mice**			
Mice with additional metastasis only found with FGS	4	50%	16; 84%

All mice (*n* = 8) developed metastasis, and in 50% of the mice additional metastases were found after turning the fluorescent camera on. FGS: Fluorescense guided surgery using ICG-Glu-Glu-AE105. Number of metastasis found during surgery.

**Figure 3:**
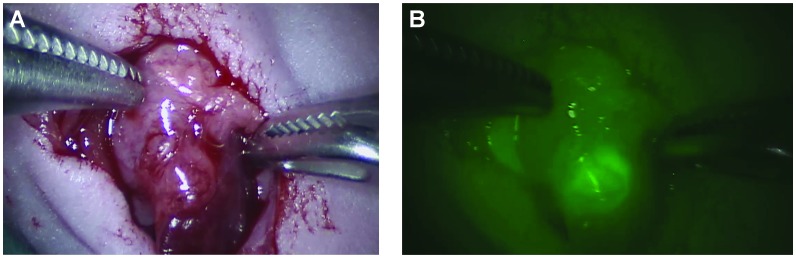
Images of a primary orthotopic pancreas human xenograft tumor as seen with the robotic Da Vinci^®^ system. This system allows the surgeon to switch between normal colour image and a fluorescent image. The image is taken 15 h post injection of ICG-Glu-Glu-AE015 after an incision in in the abdomen. (**A**) Image represent a normal white light operating view while image (**B**) is the fluorescent view with NIR vision turned on.

To explore the feasibility of NIR fluourescense-guided surgery of pancreatic cancer in a clinically relevant setup, we performed surgery in one mouse using the da Vinci^®^ HD Si surgical robotic system. The mouse was similar to the other mice in the study, with an orthotopic pancreas tumor and the procedure was performed as open surgery. After the abdomen was opened and the region of the pancreas was located, the firefly NIR fluorescence function in the robot was activated (Figure 3), and a clear fluorescent signal confined to the tumor was observed. Further, switching between NIR imaging and white light imaging in the robot to evaluate anatomy, permitted instant integration of the two modalities. The fast change between white light and fluorescent light enables an easy and intuitive guidance by the fluorescent signal along with high quality colour imaging of the anatomy to allow optimal surgical navigation (see Supplementary Video 2).

## DISCUSSION

In the present study the novel optical uPAR targeted probe ICG-Glu-Glu-AE105 was able to demonstrate clear localization of tumor deposits in an orthotopic human xenograft pancreas tumor model. Furthermore, with the optical probe a substantial number of additional metastases, not found during white light surgery, were identified. We therefore suggest, that uPAR optical guided surgery has the potential to improve outcomes in pancreatic cancer surgery.

For many years surgical oncology has relied on the surgeon’s subjective assessment on delineating tumor tissue from the surrounding healthy tissue based on the use of palpation and visual inspection under white surgical light to decide the extent of surgery. Radical resection is the primary treatment in the majority of solid tumors and presence of positive margins is of upmost importance to avoid, because even small deposit of tumor cells left behind increase the risk of recurrence. Optical guidance during surgery has therefore been introduced in the surgical field and show promising results both with untargeted and targeted optical probes. The predominant compound that has been used for passive accumulation in sentinel lymph nodes and primary tumor lesions is ICG [6]. It is well known that ICG retention in the sentinel lymph nodes is due to high affinity for albumin and therefore drainage by the lymphatic system, while the EPR effect is the main mechanism for the fluorophore to accumulate in the primary lesion. Therefore, free ICG is only suitable to detect tumors with high vascularity and is not tumor specific. Furthermore, the EPR effect is known to be highly variable [7]. For specific targeting of the tumor lesion, a receptor or surface protein highly expressed on the cancer cells is necessary for development of an agent that can accumulate and increase the fluorescent signal from tumor and metastases.

Biomarkers for cancer have been heavily studied during the last decade. Several biomarkers have shown high expression in specific cancers, which makes them potential targets for fluorescent probes for use in fluorescent guided surgery. Several fluorescent probes have been developed, to target these biomarkers, but only a few have entered clinical trials including probes targeting EGFR [9], folate [8], CEA [21] and bombesin receptors (NCT02910804). Even though these probes show promising results, tumor lesions are prone to heterogeneity and it may be of key importance to identify targets that are expressed by the majority of the tumors present in a patient group as translation into clinical use has proved difficult, time consuming and expensive, mainly due to dose challenges and regulatory requirements [22]. Even though ICG may not be an optimal fluorophore in terms of kinetic and fluorescent properties, it has the advantages of having been used extensively clinically as an intravenous agent since the 1950’s. Given the favorable safety profile of ICG and the fact that the peptide moiety AE105 has been used in more that 300 patients included in a number of clinical uPAR-PET Phase I/II trials without any side-effects [17, 18] (NCT02964988, NCT02945826, NCT02681640, NCT03278275, NCT02960724, NCT02965001 and NCT03307460), ICG-Glu-Glu-AE105 is an attractive agent for clinical translation compared with other imaging agents, where both targeting moiety and fluorophore may not have been tested in humans before. The PET probe already developed and tested in several cancer types at our department would also be useful in stratifying patients suited for targeted intraoperative optical imaging, i. e. if the uPAR PET scan is positive in the tumor/metastases, then uPAR optical guidance will be relevant. As a biomarker uPAR exhibits abilities important for use in fluorescence guided surgery. uPAR is expressed in many cancer types including breast, prostate, head & neck, glioblastoma, colorectal and pancreas cancer. Expression of uPAR is often highest at the tumor margin and in the surrounding activated tumor-associated stroma. The stroma plays a major role in pancreatic tumor, with up to 90% of the tumor volume consisting of stroma cells potentially uPAR positive [23]. A study which investigated biomarker expression in pancreatic adenocarcinomas, has shown that in 66% of patients, neoplastic cells had elevated uPAR expression while 82% had high expression in stroma associated cells [24]. This makes uPAR an ideal marker particular when the margins are uPAR positive. A fluorescent probe targeting both tumor and stroma is therefore an advantage to improve the number of negative margins and radical resections with fluorescence guided surgery.

One of the uPAR-targeted optical probes that has been investigated is the antibody based uPAR-targeted multimodal tracer ZW800-^111^In-ATN-658, which was tested in a head & neck cancer animal model [25]. However, the use of antibodies as targeting agent compared to peptides has both advantages and disadvantages. Antibodies are easier to conjugate to a fluorophore or chelator without influencing the affinity for the target. However, the major disadvantage with antibodies for optical imaging is the pharmacokinetics, where the long clearance time makes them potentially unsuitable as imaging agents compared to smaller proteins and peptides with fast kinetics and clearance from the background [26]. Antibodies have an optimal imaging time window from 24–72 h, which is impractical for surgery from a logistic point of view. This can partly be overcome by using affibodies or nanobodies as previously demonstrated [27, 28]. Affibodies still keep the specificity for the target and are easy to conjugate while the clearance time is substantially lowered compared to a full antibody. However, from a kinetic point of view peptide based ligands still may be the ideal choice. The first optical uPAR tracer was based on the aminoterminal fragment ATF of uPA [29]. This tracer showed promising results but was conjugated to the NIR fluorophore Cy5.5 that is not suitable for use in humans. Also, due to a long circulating time the imaging window was around 24 h. With these considerations in mind the optimal tracer in our view consists of a small peptide conjugated to a bright and non-toxic fluorophore.

Our results showed that the peptide based uPAR ligand ICG-Glu-Glu-AE105 tracer circulated in the blood and accumulated in the tumorous tissue to obtain a mean TBR of 3.5 in the primary lesions. This is well above the minimum of a TBR of 2 which is generally accepted necessary as a rule-of-thumb in order to effectively delineate lesions. Also, the metastases demonstrated a high TBR of 3.4. Metastases identified during fluorescent guided imaging were as small as 1 mm and still clearly visible. This demonstrate that our optical tracer together with a NIR camera is able to detect metastasis in the millimeter range and satellite branches of the tumor lesion during surgery even though not visible with the naked eye under white operation light. These findings are in concordance with our previous study that identified submillimeter tumor deposits in the neck lymph nodes in an orthotopic tongue cancer model by use of optical guidance [20]. Imaging time after injection for our optical tracer has been evaluated in a previous study [19], and was found to be high between 6 hours and up to 24 hours and is therefore very compatible with the clinical routine. Both clinically approved camera systems, Fluobeam800^®^ and the da Vinci Surgical System^®^ are sensitive enough to visualize weak signals from the tumors, while the da Vinci Surgical System^®^ has the advantage of quick switching between white light anatomy and the fluorescent signal.

The second part of our study showed that the use of fluorescence guided surgery improved surgery by identifying additional metastases in 50% of the mice. Based on total number of metastases identified, an additional 14% (95% CI: 5;28) metastases were found using the uPAR optical guidance.

In conclusion, our study showed that ICG-Glu-Glu-AE105, targeting uPAR, is a promising imaging agent for fluorescence guided surgery. With uPAR guided surgery additional metastases were found in 50% of the mice suggesting that uPAR optical guidance may lead to better surgical outcome. Our findings support clinical translation of ICG-Glu-Glu-AE105.

## MATERIALS AND METHODS

### Materials

The peptide AE105 [30] was conjugated via the α-aminogroup to ICG and purchased from ABX (Radeberg, Germany) [19]. For *in vivo* injection ICG-Glu-Glu-AE105 was dissolved in (2-hydroxypropyl)-β-cyclodextrin with 2% DSMO. The human pancreas adenocarcinoma cell line BxPC3-luc2 was purchased from PerkinElmer and grown after the recommendations in RPMI-Glutamax with 10% FBS and 1% PenStrep.

### Animal model

All animal experiments were performed under a protocol approved by the Animal Research Committee of the Danish Ministry of Justice (2016-15-0201-00920). The mice were anesthetized with 4% sevoflurane before an incision in the abdomen was made over the spleen and the pancreas was located. 5 × 10^5^ BxPC3-luc2 in 25 ml PBS was injected orthotopic in the tail of the pancreas. The incision was closed with 5.0 suture. The primary tumor was followed by luminescence and when a suitable size was reached a similar incision was made and the primary tumor was removed and the incision was closed again as described above. During the study all mice were housed in groups with 12 h light/dark cycle with food and water available ad libitum.

### Camera

The FluobeamR800 (Fluoptics, Grenoble, France) used for navigation during resection of the primary tumor and the metastases is a handheld camera with an excitation wavelength at 800 nm where adjustments as sensitivity and zoom are possible. We used the clinically approved surgical robot system da Vinci^®^ HD Si, (Intuitive Surgical, California, USA) using non-human instruments, in one case for navigation in resection of a primary human xenograft tumor in the pancreas of a mouse. IVIS Lumina XR, a black-box optical camera was used for luminescence imaging for growth monitoring of the tumor and positive control for tumor presence in the resected metastases.

### Part I – feasibility of optical imaging in an orthotopic pancreas model

After inoculation of BxPC3-luc2 cells in the pancreas as described above the mice were luminescence imaged to follow the growth of the primary tumor. When the primary tumor reached a suitable size of approximately 50–100 mm^3^, 10 nmol of ICG-Glu-Glu-AE105 was injected i.v. into the mouse 15 h pre-operatively. The primary tumor was then removed and a fluorescent image of the tumor was performed throughout the surgery. Because it is known that removal of the primary tumor does not prevent metastases in the peritoneum from developing, the incision was closed and the mice underwent luminescence imaging to track the development of potential metastases. When potential metastatic burden was assessed in the abdomen, an incision was again made in the abdomen and the fluorescent camera was turned on to locate and image the metastases developed. TBR was later measured using ImageJ software, where a ROI was drawn over the whole tumor and a background ROI was drawn over adjacent tissue.

### Part II – white light guidance compared to fluorescent guidance

**Figure 4:**
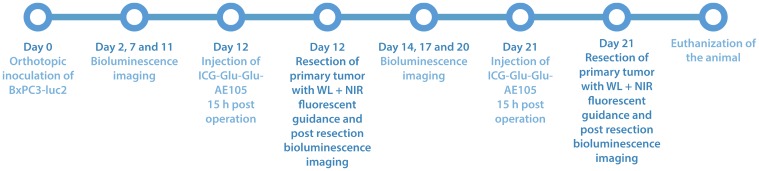
Study timeline over part II of the study, where white light surgery is compared with fluorescent guided surgery in 5 mice with ICG-Glu-Glu-AE105 was used as optical probe 15 h post iv. injection. The tumor growth was followed with bioluminescence before surgery to ensure tumor size and spread and was done after surgery to ensure that residual tumor foci were located after surgery.

The same work flow as in study I, except that the surgeon was asked to remove all the metastases he could identify with regular white light turned on during surgery. When the surgeon was convinced all metastases were removed the NIR camera was used to identify potentially additional metastases. The mouse was then imaged with bioluminescence to locate further metastases that was missed by both white light and NIR light (Figure 4). All metastases were imaged *ex-vivo* to validate the presence of tumor cells in the tissue assumed to be a metastasis. The mice were euthanized after the operation.

### Statistical methods

Data are described as mean and 95% confidence interval.

## SUPPLEMENTARY MATERIALS







## Abbreviations

ATF: amino terminal fragment
CEA: carcinoembryonic antigen
EGFR: epidermal growth factor receptor
EPR: enhanced permeability and retention
FGS: fluorescence guided surgery
ICG: indocyanine green
NIR: near infrared
PET: positron emission tomography
TBR: tumor to background ratio
uPA: urokinase plasminogen activator
uPAR: urokinase plasminogen activator receptor
WL: white light
