# Supplementary material for: Improved surgical resection of metastatic pancreatic cancer using uPAR targeted in vivo fluorescent guidance: comparison with traditional white light surgery
Source: Oncotarget. 2019 Oct 29;10(59):6308–16. doi: 10.18632/oncotarget.27220 (PMC6824874; doi:10.18632/oncotarget.27220)
Supplement: Supplementary file 1 [file oncotarget-10-6308-s001.pdf]

Improved surgical resection of metastatic pancreatic cancer using uPAR targeted *in vivo* fluorescent guidance: comparison with traditional white light surgery

SUPPLEMENTARY MATERIALS

Supplementary Video 1: Metastatic surgery guided by the fluorescent signal. See Supplementary Video 1

Supplementary Video 2: Localization of a primary pancreatic human xenograft tumor using our fluorescent probe together a da Vinci R HD Si robotic system. See Supplementary Video 2
